# Reshaping Faces, Redefining Risks: A Systematic Review of Orthognathic Surgery Outcomes in Cleft Lip and Palate Patients

**DOI:** 10.3390/jcm13195703

**Published:** 2024-09-25

**Authors:** Sachin R. Chinta, Sergio Segrera, Rebecca Friedman, Alay R. Shah, Rami S. Kantar, Angela S. Volk, David Staffenberg, Eduardo D. Rodriguez

**Affiliations:** Hansjörg Wyss Department of Plastic Surgery, New York University Langone Health, New York, NY 10016, USA

**Keywords:** orthognathic surgery, cleft lip and palate, risk management, surgical outcomes

## Abstract

**Background:** This study aims to determine a generalized outcome and risk profile for patients undergoing orthognathic surgery for the definitive treatment of cleft lip and palate. Furthermore, we hope to determine the key risk factors that cause increased risk for cleft lip and palate patients undergoing orthognathic surgery. **Methods:** This study includes a systematic review using PubMed, MEDLINE, Cochrane, and Scopus. Data curation utilized Covidence software, with dual-reviewer screening and conflict resolution by a third party, focusing on publications with the full texts available. **Results:** The initial search yielded 1697 articles. Following title, abstract, and full-text screening, a total of 62 articles were included in this review. A total of 70.9% of included articles had moderate bias, with the rest having low risk of bias. The sample consisted of 2550 patients with an average age of about 20 years and an average follow-up of 16.8 months. The most employed procedure was Le Fort I osteotomy (99%). In terms of velopharyngeal function, there were notable increases in insufficiency and severity scores, with an average 63% worsening score from the baseline. That being said, patients experienced an average 33% improvement in speech articulation. Furthermore, the average horizontal movement was reported to be 6.09 mm with a subsequent relapse of 0.98 mm overall. **Conclusions:** This systematic review distills data from 62 articles and 2550 patients. It highlights the efficacy of orthognathic surgery in addressing oropharyngeal and aesthetic deficits. This study identifies relapse and velopharyngeal insufficiency as recurrent complications. These insights inform surgical refinement and patient counseling, laying a foundation for enhanced clinical protocols.

## 1. Introduction

Patients with cleft lip and palate (CLP), an ailment that affects 1 in 700 live births globally, can often experience a broad spectrum of impairments that affect both function and aesthetics [1]. CLP is a congenital condition where the tissues of the upper lip and/or roof of the mouth do not fully fuse during fetal development, ultimately leading to speech difficulties due to velopharyngeal insufficiency, dental malocclusion, and facial asymmetry. Taken together, these deficits can lead to significant psychosocial challenges, such as reduced self-esteem and social stigmatization [2]. Primary surgical repair of CLP may produce soft-tissue changes that can restrict maxillary growth, resulting in midface hypoplasia and class III occlusion. Approximately 25% of patients with unilateral CLP patients develop significant maxillary hypoplasia leading to a loss of facial height, pseudoprognatism, and severe dental malocclusion [3,4]. As a result, orthognathic surgery (OS) frequently serves as the culminating phase of a comprehensive treatment strategy for CLP patients.

Correction of class III malocclusion is typically done with maxillary advancement using conventional Le Fort I advancement with or without a mandibular setback. In patients with CLP, however, soft-tissue contracture and scar formation lead to higher rates of relapse and post-operative instability when compared to those without cleft [5,6,7]. Additionally, maxillary advancement can have a significant effect on soft-palate musculature and structure post-operatively. Alterations to the velopharyngeal mechanism in these patients carries an increased risk of velopharyngeal insufficiency and the development of hypernasal speech [8]. Taken together, correction of midface hypoplasia in patients with a cleft should not be undertaken without considering the aforementioned implications to functional speech, relapse, facial aesthetics, and ultimately quality of life.

While previous reviews have focused on specific metrics of post-operative success following orthognathic surgery, most have been limited in their scope and follow up period. We aim to holistically evaluate the functional and structural outcomes of conventional orthognathic surgery in patients with CLP. In the present report, we aim to utilize the largest review of the literature on orthognathic CLP to date to evaluate key longitudinal metrics that could impact established evidence-based directives.

## 2. Methods

### 2.1. Search Strategy and Information Sources

A systematic review was conducted in accordance with the Preferred Reporting Items for systematic Review and Meta-Analyses (PRISMA) 2020 guidelines using the PubMed, MEDLINE, Cochrane, Embase, and Web of Science databases without a restriction on time of publication. Databases and registers were accessed and queried in December 2023. Search terms related to OS and cleft lip and/or palate were used for the literature search. A complete list of search terms can be found in Table 1. Following identification, the relevant studies were uploaded into Covidence (Covidence systematic review software, Veritas Health Innovation, Melbourne, Australia, 2023 available at www.covidence.org) to aid in the review process and reduce any potential sources of conflict or bias. 

### 2.2. Inclusion and Exclusion Criteria

Criteria for inclusion and exclusion are outlined in Table 2. Title, abstract, and full-text screens were conducted by two independent reviewers (S.S., R.F.). All conflicts were resolved by a third-party reviewer (S.R.C.). Due to the relative abundance of studies, abstracts with no full text were excluded from the review. Notably, only studies involving non-syndromic patients undergoing orthognathic surgery following cleft lip and/or palate repair were included. Additionally, studies involving distraction osteogenesis were excluded to minimize confounding effects and avoid introducing any complications specific to that procedure.

To assess the risk of bias of the studies included in this review, we utilized the Cochrane ROBINS-I tool, which is specifically designed to evaluate non-randomized studies of interventions. Each study was evaluated independently by two reviewers (S.S., R.F.), with disagreements resolved by a third reviewer (S.R.C.). The tool classifies the risk of bias across seven domains, including confounding, selection of participants, classification of interventions, deviations from intended interventions, missing data, measurement of outcomes, and selection of reported results. Only studies deemed to have a “low” or “moderate” risk of bias were included in our analyses to ensure the reliability of the conclusions. Per Cochrane ROBINS-I guidelines, studies judged to have a “low” risk of bias were assessed to have a low risk of bias across all domains. Studies judged to have a moderate risk of bias in at least one domain, and not assessed to have a high risk in any domain, were classified as “moderate” risk of bias.

### 2.3. Data Collection Process

Data extraction was performed by three independent reviewers (S.S., R.F., S.R.C.) and included year of publication, patient demographics, surgical technique, operative outcomes (velopharyngeal, stability/relapse, cephalometric), and complications. If not explicitly documented in the article, data were marked as not reported. All measures, time points, and analyses for each relevant post-surgical outcome were included in the data extraction. Data of control groups involving patients without cleft lip and/or palate were not included in the extraction.

To evaluate velopharyngeal, airway, and speech outcomes, individual results were tabulated for each study. Outcomes that were reported by at least 4 studies were selected for synthesis. Summary statistics were calculated by finding the average percent change from baseline. For outcomes utilizing composite scores to assess post-operative changes, including velopharyngeal function/insufficiency and hypernasality, percent change from baseline was calculated for each score given the heterogeneity in the scales used. For variables with binary outcomes (i.e., present or not present), the mean change of percentage of patients was calculated.

For cephalometric outcomes, maxillary position at pre-operative evaluation was compared to maxillary position at the immediate post-operative visit to determine surgical advancement. Relapse was then defined as the change in position assessed at a 12-month follow-up relative to the position noted at the immediate post-operative visit. Cephalometric angular changes and relapses were assessed similarly. Immediate post-operative evaluation included assessments completed no later than 6 weeks after surgery. Movements in the horizontal plane were denoted as positive when the maxilla was repositioned anteriorly and negative when moved posteriorly; in the vertical plane, downward repositioning was denoted as positive and upward movement was negative. When specified, movements were in reference to the A point (the deepest point on the midline of the maxillary alveolar bone). Many studies included mean advancements and relapses in their analyses. For studies that did not give mean changes for a specific outcome but did note pre-operative and post-operative values, mean change was calculated by a study member. Studies that that did not include post-operative evaluation values were excluded from the overall analysis. Studies with a follow-up period of less than 12 months were not included for relapse analysis but were included for surgical change analysis if pre-operative and post-operative values were included.

## 3. Results

**Figure 1:**
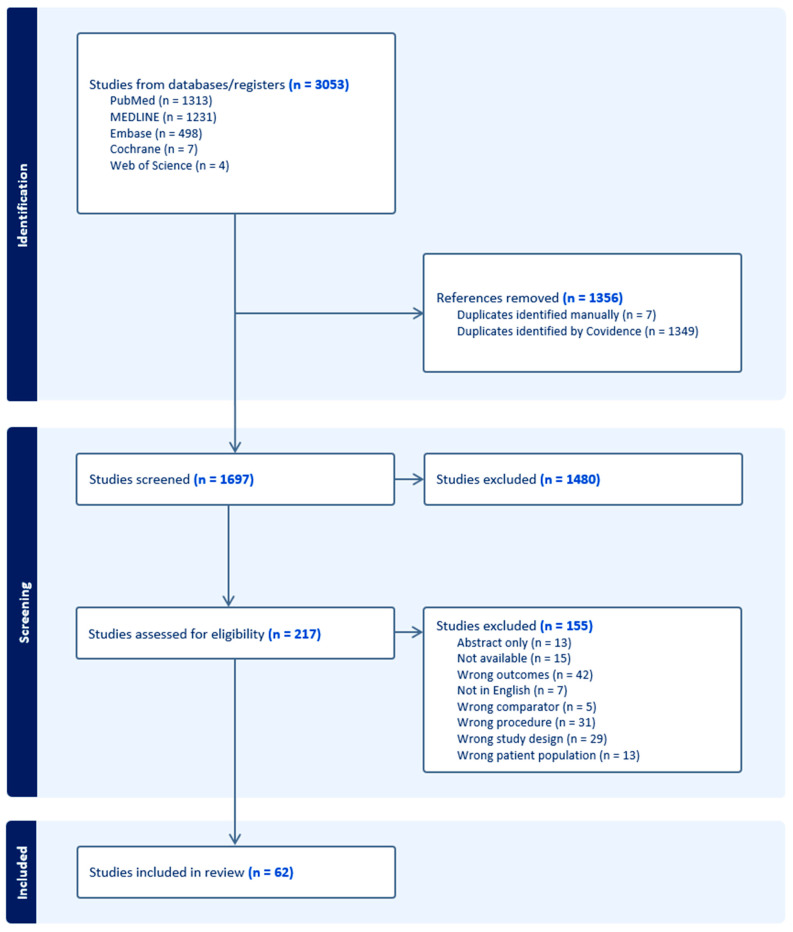
PRISMA diagram.

The PRISMA flow chart is presented in Figure 1. Briefly, 3053 studies were exported from databases and registers, of which 1697 abstracts were screened. Of these, 217 full texts were assessed for eligibility. In total, 62 studies were included, ranging in publication from 1979 to 2023, with more than half (35) being published in the last ten years. Using the ROBINS-I tool, we assessed the risk of bias across the included studies (Supplementary Table S1). In total, 70.9% of studies were classified as having a “moderate” risk of bias, primarily due to confounding variables and the measurement of outcomes. The remaining studies (29.1%) had a “low” risk of bias. Several studies did not account for baseline differences in patient characteristics or prior surgical history, which could introduce bias due to confounding. Additionally, bias in the measurement of outcomes was observed in studies with non-standardized evaluation of velopharyngeal function and speech outcomes.

### 3.1. Demographics

The included studies surveyed 2550 patients with an average pre-operative age of 20 years. Study demographics are presented in Table 3 [5,9,10,11,12,13,14,15,16,17,18,19,20,21,22,23,24,25,26,27,28,29,30,31,32,33,34,35,36,37,38,39,40,41,42,43,44,45,46,47,48,49,50,51,52,53,54,55,56,57,58,59,60,61,62,63,64,65,66,67,68,69]. Gender was reported for 87 percent of patients, of which the majority (57%) were male. Regarding the preliminary cleft repair, 1583 (62%) patients had a unilateral cleft lip and palate, 616 (24%) had a bilateral cleft lip and palate, and 212 (8%) had an isolated cleft palate (cleft type was not reported for 139 patients).

Most studies (81%) assessed one group of patients over time (Table 4), while 19% of studies compared groups of patients according to cleft type, surgical technique, and other clinical characteristics (Table 5). A total of 2517 (99%) patients received a Le Fort I osteotomy and 906 (36%) of these underwent mandibular osteotomy as well (bimaxillary osteotomy). Additional data on prior and concurrent procedures are detailed in Table 4. Similar information for studies without comparator groups can be found in Table 5. Most studies (55%) examined changes in velopharyngeal function, speech, and airway dimensions, and many (50%) also examined cephalometric changes and stability after surgery. The average length of follow-up after surgery was 16.8 months (ranged from 0.7 to 66 months), but most commonly (35%), patients were followed for a 12-month period.

### 3.2. Velopharyngeal and Speech Outcomes

Velopharyngeal and soft-tissue outcomes are reported in Table 6 and Supplementary Table S2. In terms of velopharyngeal function, there was a notable increase in insufficiency and severity scores, with an average 63% worsening of the scores from baseline. The data also indicate changes in pharyngeal and airway dimensions, with an average 13% increase in cross-sectional and volume measurements post-operatively. Additionally, the pharyngeal depth generally increased, with a 17% change from baseline. Various studies reported on changes in nasal dimensions with mixed outcomes.

Speech and airway outcomes are reported in Table 6 and Supplementary Table S3. The data compiled show a variety of outcomes, with several studies noting an average 15% increase in hypernasality or resonance severity. Similarly, there was an average 20% worsening in nasal emission or turbulence compared to baseline. Results of speech articulation or intelligibility post-operatively were mixed; however, most reported a decrease in articulation errors with an average 33% improvement.

### 3.3. Cephalometric Outcomes

Outcomes on cephalometric changes and stability can be found in Table 7 and Supplementary Tables S4–S6. On average, SNA angle increased by 5.88 degrees and relapsed by 1.27 degrees across all included studies. When stratified by surgical technique, we found that studies with patients who received only a Le Fort I osteotomy had an average surgical change of 6.11 degrees and a relapse of 1.13 degrees. One study in which all patients underwent a Le Fort II osteotomy showed an average change of 6.10 degrees. SNB angle decreased by 1.27 degrees and relapsed by 0.90 degrees overall, and when separated into the same surgical groups showed a decrease of 1.32 degrees and 0.80 degrees, respectively. An overall increase of 7.54 degrees and a relapse of 3.51 degrees were found for the ANB angle. According to surgery type, we found a mean increase in the ANB angle of 7.82 degrees and 5.00 degrees and a mean relapse of 3.22 and 6.00 for Le Fort I and Le Fort II osteotomies, respectively.

For maxillary surgical advancement, we found the average horizontal movement to be 6.09 mm overall. The average horizontal advancement was 6.58 mm in studies with patients who only underwent Le Fort I osteotomy compared to 3.95 mm in studies with patients who all received a bimaxillary osteotomy. The subsequent relapse was found to be 0.98 mm overall, with a mean of 0.97 mm and 0.52 mm in studies with patients who received only a Le Fort I osteotomy versus a bimaxillary procedure, respectively. In the vertical plane, the overall mean advancement was 3.28 mm and the overall relapse was 0.89 mm. The mean vertical advancement in studies with only Le Fort I patients was 3.07 mm, and relapse was 0.74. For studies where all patients underwent bimaxillary osteotomies, the mean vertical advancement was 3.26 mm, and relapse was 0.86 mm.

## 4. Discussion

The outcomes of OS in patients who have undergone primary repair of CLP are pivotal not only for facial aesthetics but also for functional rehabilitation. That being said, the role OS has on impacting facial cephalometry, velopharyngeal function, and speech articulation is unclear. This impacts a provider’s ability to deliver valuable counseling on these critical aspects of comprehensive patient care. With this analysis, we aimed to elucidate the extent to which corrective jaw surgery can address the underlying skeletal deformities inherent to CLP, improve velopharyngeal competence for speech, and ultimately enhance speech intelligibility and quality.

### 4.1. Velopharyngeal and Speech

Velopharyngeal insufficiency (VPI) is not seen as a definitive complication following primary palatoplasty due to the confounding natural incidence of soft-tissue insufficiency in cleft populations. Issues with cleft width, tissue availability, and palatal dimensions are highly variable and it can be difficult to correct them in a single operation [70]. Additionally, it is important to note that correcting VPI may or may not correct issues with speech articulation. The muscular function of the soft palate constitutes only one aspect of speech development, and this is demonstrated by palatoplasty improving articulation independent of VPI status [70]. Furthermore, maxillary advancement following the primary repair of CLP compounds the complexity of this clinical picture.

The sagittal advance of the maxilla has controversial effects on velopharyngeal function, particularly in the context of CLP. Previous reports have postulated that patients with CLP are at a higher risk for velopharyngeal deficiency due to issues with scarring limiting the adaptation of the velopharyngeal muscles post-operatively and due to a compensatory increase in pharyngeal depth. Our own review noted that in the studies presenting pre- and post-operative velopharyngeal data, there was an average increase of 63% in velopharyngeal dysfunction/insufficiency on the composite scores used to assess this outcome. Importantly, this does not signify the true rate of VPI post-operatively but instead points towards an overall change in function following surgical advance. Regardless, this, considered in conjunction with an average increase of 13% and 17% in pharyngeal volume and depth, respectively, calls the safety, as it pertains to soft-tissue outcomes, of current advancement techniques into question [8,9,71]. This is due to the documented effect that changes in velopharyngeal function, depth, and volume can also have on a patient’s swallowing ability, as well as on their protective airway mechanism [72]. These data highlight the need for extensive pre-operative counseling for patients on the risks associated with corrective OS. Furthermore, OS in the setting of CLP should be conducted in conjunction with a trained speech—language pathologist, whenever possible. Early intervention with speech pathology could provide functional benefits that are necessary in the post-operative course of any CLP patient. Of note, there is a lack of standardization in regard to evaluating velopharyngeal function after corrective OS. Studies have suggested various techniques, including lateral cephalograms aimed at assessing pharyngeal dimensions or qualitative surveys scored by the operative clinician, but this lack of homogeneity precluded any advanced statistical analysis in this review. Additionally, the absence of a consensus on VPI assessment tools makes parsing the relationship between maxillary advancement, velopharyngeal dysfunction, and speech challenging.

Speech outcomes following maxillary advancement are dependent on various factors, many of which cannot be controlled for in a single-staged operation. In the setting of CLP, speech outcomes following conventional OS have demonstrated inter-study inconsistency, with reports of both increases and decreases in speech articulation post-operatively. A point of consensus, however, is that patients with CLP are much more likely to develop difficulties with speech articulation when compared with healthy controls following OS [9]. Among the studies that reported changes in articulation, our review found there to be an average improvement of 33% on composite scoring surveys used by providers. Conversely, we identified an average worsening of 15% and 20% for hypernasality and nasal emission, respectively, following OS in CLP populations. This does not necessarily stand in opposition to previously published reports that have detailed similar findings in terms of nasality and speech articulation in operated CLP patients, but the etiology of this constellation of symptoms has yet to be defined [42]. Furthermore, the role of impaired soft-palate musculature as opposed to maxillary advance and its direct impact on speech emission and articulation has been sparsely researched. It is possible to delineate which of the parameters has a greater impact on speech post-operatively using nasofibroscopic evaluation, but previous work utilizing this technique lacks standardization, which precludes meaningful comparison [8,73,74]. We recommend prospective studies utilizing direct pharyngeal visualization to evaluate the relationship between maxillary advance and soft-tissue changes as it relates to its synergistic or antagonistic effect on speech post-operatively.

### 4.2. Cephalometric

When comparing patients with unrepaired unilateral or bilateral CLP, previous research has identified that these patients classically develop a protruding, or prognathic, maxilla [75,76]. CLP patients with prior surgical intervention, alternatively, present with significantly higher rates of midface hypoplasia when compared to unoperated cohorts. While a well-developed understanding of maxillary growth mechanics following surgical disruption is lacking, the previously mentioned findings underpin the understanding that a primary surgical correction of CLP may be the etiology of midface hypoplasia within operated cohorts. Regardless of the cause, the correction of a hypoplastic maxilla with OS often relies on pre- and post-operative cephalometric analysis to ensure adequate and stable reconstruction [77].

The decision to undergo OS for the correction of midface hypoplasia is often based on the clinical evaluation of sagittal head films that assess cephalometric angles, including SNA, SNB, and ANB. SNA, which is an orthodontic measure used to assess the relationship between the cranial base and the maxilla, has been documented as being lower on average in operated CLP patients when compared to healthy controls [77]. Aesthetically, this manifests as deficient anterior projection of the upper jaw, while also limiting functional dental occlusion in CLP patients. Our review identified an average increase of 5.88 degrees in SNA, as well as an average horizontal advance of 6.09 mm, in patients included in this review. This, taken together with an average decrease of 1.27 degrees in SNB, points toward the efficacy of OS in correcting midface hypoplasia in CLP patients. This is further supported by an average increase in ANB of 7.54 degrees. That being said, the long-term stability of these results is of competing importance as operative results must prove to be steadfast and reliable.

### 4.3. Relapse

Skeletal relapse following OS in CLP patients has been shown to manifest at a significantly higher rate in the cleft population when compared to healthy controls [20]. The reason for this is likely multifactorial, but palatal scarring, soft-tissue tension, and areas of patchy vascularity from index-repair operations have all been identified as contributors to increased rates of relapse. Relapse is particularly concerning because, dependent on the degree, instability can necessitate reoperation to establish a proper occlusive relationship between the upper and lower jaw. Previous studies have identified the degree of relapse requiring reoperation to be >4 mm, whereas <2 mm has been deemed within the method of error and is not considered to be clinically significant. Relapse within 2–4 mm, alternatively, is treated as clinically significant but can be overcome by classic dental compensation [78]. Our review identified an average relapse of 0.98 mm in the horizontal dimension and 0.89 mm in the vertical dimension. While there was a degree of skeletal relapse in every vector, average relapse was determined to be within the method of error and can be overcome by post-operative orthodontics if clinically relevant. That being said, in clinical scenarios where there is increased suspicion for the development of skeletal relapse, technique modification, as well as alterations in post-operative management, can be employed. Modifying classic Le Fort techniques with adjuncts such as down-grafting or segmental osteotomies can lead to decreased relapse when paired with appropriate bony fixation. Additionally, enacting close follow-up to monitor bone healing for stable occlusion is essential in situations with a high probability of bony relapse.

## 5. Limitations

A notable limitation of this review arises from the inherent confounding factors, such as the severity of the initial cleft conditions, prior surgical interventions, and individual anatomical differences, which can significantly influence surgical outcomes but may not be fully accounted for in the analyzed studies. Craniomaxillofacial growth, in particular, is something that could have impacted post-operative cephalometric analysis. Additionally, the lack of standardization in outcome measures across studies presents a substantial challenge. Specifically, the varied methods used to assess VPI and speech improvements result in a heterogeneity that complicates the comparison of results and the synthesis of conclusive findings. This diversity in measurement and reporting standards underscores the need for more uniform assessment protocols to enhance the reliability and comparability of future research in this field. The strength of this study lies in it being the largest review of the literature pertaining to OS following primary CLP repair to date, as well as in the comprehensive review we offer of cephalometric, velopharyngeal, and speech outcomes following OS in CLP populations.

## 6. Conclusions

In conclusion, our systematic review provides a comprehensive analysis of the functional and structural outcomes of orthognathic surgery in patients with a history of primary cleft lip and palate repair. The findings underscore the pivotal role of maxillary advancement in correcting midface hypoplasia and enhancing facial aesthetics in cleft lip and palate populations. Despite these positive outcomes, our review also highlights the inherent challenges, such as relapse, and the complexity of managing velopharyngeal insufficiency post-operatively. These issues necessitate a multidisciplinary approach to ensure optimal patient care. Moving forward, there is a clear need for long-term studies with standardized outcome measures to better understand the durability of surgical results and the comprehensive impact of orthognathic surgery on speech and velopharyngeal function in these populations. By integrating these insights into clinical practice, we can refine surgical techniques and improve pre-operative planning to maximize the benefits of orthognathic surgery for cleft lip and palate patients.

## Figures and Tables

**Table 1:** Search criteria for literature search.

Orofacial	AND	Orthognathic
Cleft Palate [Mesh]		Orthognathic Surgery [Mesh]
OR		OR
Cleft Lip [Mesh]		Orthognathic Surgical Procedures [Mesh]
OR		OR
Orofacial Cleft [Mesh]		Mandibular Osteotomy [Mesh]
		OR
		Maxillary Osteotomy [Mesh]
		OR
		Osteotomy, Sagittal Split Ramus [Mesh]
		OR
		Osteotomy, Le Fort [Mesh]
		OR
		Osteotomy [Mesh]

**Table 2:** Inclusion and exclusion criteria.

	Inclusion	Exclusion
Article type	Randomized controlled trials (RCTs), cohort studies, case–control studies, and cross-sectional studies, case series (n > 5)	Case series (n < 5), case reports, editorials, commentaries, review articles, unpublished data, and conference abstracts
Population	Non-syndromic human subjects	Animal studies, syndromic subjects
Interventions	Orthognathic surgery following cleft lip and/or palate repair, including Le Fort osteotomies (single- or multi-piece), mandibular osteotomies, genioplasties, sagittal split osteotomy	Distraction osteogenesis
Language	English	Non-English
Data	Readily available data	Insufficient data or inadequate methodology

**Table 3:** Study demographics.

	Total Patients	Average Age (years)	Female # (%)	Male # (%)	Cleft Type			
					CP/SMCP # (%)	UCLP # (%)	BCLP # (%)	Other # (%)
Schendel 1979 [9]	8	27	NR	NR	-	-	-	CLP: 8 (100)
Ward-Booth 1984 [10]	13	20.4	NR	NR	-	7 (54)	6 (46)	-
Adlam 1989 [11]	27	21.1	13 (48)	14 (52)	-	18 (67)	9 (33)	-
Houston 1989 [12]	30	17.25	19 (63)	11 (37)	1 (3)	24 (80)	5 (17)	-
Posnick 1990 [13]	30	18	NR	NR	-	30 (100)	-	-
Watzke 1990 [14]	24	17.1	9 (38)	15 (62)	-	20 (83)	4 (17)	-
Hochban 1993 [15]	14	26.1	6 (43)	8 (57)	-	14 (100)	-	-
Okazaki 1993 [16]	10	19.5	4 (40)	6 (60)	1 (10)	7 (70)	2 (20)	-
Cheung 1994 [17]	46	22	19 (41)	27 (58)	-	30 (65)	16 (35)	-
Posnick 1994 [94(1)] [18]	14	19	NR	NR	14 (100)	-	-	-
Posnick 1994 [94(7)] [19]	35	18	NR	NR	-	35 (100)	-	-
Ayliffe 1995 [20]	61	19	27 (44)	34 (56)	-	46 (75.4)	15 (24.5)	-
Stewart 1996 [21]	24	NR	12 (46)	13 (54)	-	13 (54) [46% L; 54% R]	6 (25)	CLP: 5 (20)
Haapanen 1997 [22]	15	23.3	NR	NR	-	10 (66)	5 (33)	-
Maegawa 1998 [23]	40	20.5	17 (42)	23 (58)	1 (3)	26 (65)	13 (32)	-
Heliövaara 2001 [24]	40	23.7	13 (34)	27 (66)	-	40 (100) [63% L; 37% R]	-	-
Hirano 2001 [25]	58	19.8	28 (48)	30 (52)	-	42 (72)	16 (28)	-
Heliövaara 2002 [26]	25	25.6	13 (52)	12 (48)	14 (56)	-	11 (44)	-
Trindade 2003 [27]	29	21.5	12 (41)	17 (59)	4 (14)	18 (62)	7 (24)	-
Heliövaara 2004 [28]	50	25	21 (42)	29 (58)	11 (22)	30 (60)	9 (18)	-
Janulewicz 2004 [29]	54	8–33	17 (31)	37 (69)	6 (11)	27 (50) [85% L; 15% R]	21 (39)	-
Niemeyer 2005 [30]	42	16–41	18 (44)	24 (56)	9 (21)	23 (55)	10 (24)	-
Thongdee 2005 [31]	30	18	21 (70)	9 (30)	-	30 (100)	-	-
Wolford 2008 [32]	12	12.5	3 (25)	9 (75)	-	6 (50)	6 (50)	-
Kim 2012 [33]	8	NR	NR	NR	8 (100)	-	-	-
Kumari 2013 [34]	9	17.2	5 (56)	4 (44)	-	7 (78)	2 (22)	-
Pereira 2013 [35]	20	20.2	4 (20)	16 (80)	-	-	-	CLP: 20 (100)
Davidson 2014 [36]	11	18.1	NR	NR	-	6 (55)	5 (45)	-
Watts 2014 [37]	30	18.3	NR	NR	-	30 (100)	-	-
Karabekmez 2015 [38]	15	18	4 (27)	11 (73)	1 (7)	8 (53) [63% L; 37% R]	6 (40)	-
Park 2015 [39]	25	24.1	NR	NR	-	20 (80)	5 (20)	-
Watts 2015 [5]	30	8.7	NR	NR	-	30 (100)	-	-
Wu 2015 [40]	47	20.75	NR	NR	-	33 (70)	14 (30)	-
Chang 2017 [41]	18	19.72	9 (50)	9 (50)	-	18 (100)	-	-
Impieri 2018 [42]	47	20.7	28 (60)	19 (40)	5 (11)	24 (51)	18 (38)	-
Jeong 2018 [43]	41	21.49	15 (37)	26 (63)	NR	NR	NR	NR
Alaluusua 2019 [44]	100	17.9	54 (54)	64 (64)	23 (23)	53 (53)	24 (24)	-
Hagberg 2019 [46]	15	19.6	5 (33)	10 (66)	-	7 (47)	8 (53)	-
Harjunpää 2019 [47]	93	18	51 (55)	42 (45)	25 (27)	46 (49)	22 (24)	-
De Medeiros-Santana 2019 [45]	52	23.7	NR	NR	3 (6)	38 (73)	11 (21)	-
Schultz 2019 [48]	18	19.2	9 (50)	9 (50)	-	17 (95)	1 (5)	-
Yatabe-Ioshida 2019 [49]	15	28.7	5 (33)	10 (67)	-	9 (60) [56% L; 44% R]	6 (40)	-
Alaluusua 2020 [50]	59	17.9	35 (59)	24 (41)	12 (20)	31 (53)	16 (27)	-
Ganske 2020 [51]	19	18	8 (42)	11 (58)	-	19 (100)	-	-
Pereira 2020 [52]	20	20.2	4 (20)	16 (80)	1 (5)	15 (75) [67% L; 33% R]	4 (20)	-
Saleh 2020 [53]	23	20.6	6 (26)	17 (74)	4 (9)	13 (30) [62% L; 38% R]	6 (14)	-
Susarla 2020 [31(5)] [54]	28	18.9	11 (39)	17 (61)	-	-	-	CLP: 28 (100)
Susarla 2020 [49(4)] [55]	25	18.4	NR	NR	-	25 (43)	-	-
Ho 2021 [56]	17	20.4	3 (18)	14 (82)	-	-	-	CLP: 17 (100)
Parikh 2021 [57]	35	20.65	11 (31)	24 (69)	-	25 (71)	10 (29)	-
Wangsrimongkol 2021 [58]	49	19.5	13(26)	36 (73)	-	28 (57)	21 (43)	-
Harjunpää 2022 [59]	57	17.9	33 (58)	24 (42)	12 (21)	30 (53)	15 (26)	-
Jang 2022 [69]	19	22	10 (53)	9 (47)	3 (16)	14 (77)	2 (11)	-
Seixas 2022 [60]	535	24.4	218 (41)	317 (59)	32 (6)	316 (59)	187 (35)	-
Tekin 2022 [61]	10	20.5	3 (30)	7 (70)	-	-	-	CLP: 10 (100)
Tsang 2022 [62]	17	20.4	3 (18)	14 (82)	-	17 (100)	-	-
Wangsrimongkol 2022 [63]	51	19.3	13 (25)	38 (76)	-	30 (59)	21 (41)	-
Mansour 2023 [66]	24	19.02	12 (50)	12 (50)	-	24 (100) [67% L; 33% R]	-	-
May 2023 [67]	10	18	5 (50)	5 (50)	NR	NR	NR	NR
Idso 2023 [64]	30	18.5	17 (57)	13(43)	-	30 (100)	-	-
Liao 2023 [65]	35	18	13 (37)	22 (63)	-	35 (100) [74% L; 26% R]	-	-
Su 2023 [68]	162	19	80 (49)	82 (51)	22 (13.6)	89 (54.9)	51 (31.5)	-

CP/SCMP: cleft palate/submucous cleft palate; UCLP: unilateral cleft palate; BCLP: bilateral cleft palate; NR: not reported.

**Table 4:** Methodology of included studies without comparator groups (50 studies).

	Follow-Up Period (Average Months)	Le Fort I # (%)	Le Fort II # (%)	“Bimaxillary Osteotomy # (%)”	Prior/Concurrent Procedures # (%)
Schendel 1979 [9]	26	8 (100)	-	-	-
Ward-Booth 1984 [10]	22	-	13 (100)	-	-
Adlam 1989 [11]	22	23 (85)	4 (15)	14 (52)	-
Houston 1989 [12]	17	30 (100)	-	-	-
Posnick 1990 [13]	24	30 (100)	-	3 (10)	“Pharyngoplasty/Flap: 6 (20) [Prior] Genioplasty: 12 (40) [Concurrent]”
Watzke 1990 [14]	12	24 (100)	-	-	Bone Graft: 24 (100)
Hochban 1993 [15]	12	14 (100)	-	-	-
Okazaki 1993 [16]	12	9 (90)	1 (10)	8 (80)	Pharyngoplasty/Flap: 1 (10) [Prior]
Posnick 1994 [94(1)] [18]	30	14 (100)	-	4 (29)	“Pharyngoplasty/Flap: 6 (43) [Prior] Bone Graft: 10 (71) [Concurrent]”
Posnick 1994 [94(7)] [19]	12	35 (100)	-	11 (31)	“Pharyngoplasty/Flap: 13 (37) [Prior] Genioplasty: 22 (63) [Concurrent]”
Haapanen 1997 [22]	12	15 (100)	-	-	Bone Graft: 15 (100) [Prior]
Maegawa 1998 [23]	8.4	40 (100)	-	7 (18)	Pharyngoplasty/Flap: 14 (35)
Heliövaara 2001 [24]	12	40 (100)	-	-	“Pharyngoplasty/Flap: 7 (18) [Prior] Bone Graft: 33 (83) [Prior] Rhinoplasty: 35 (88) [Prior] Lip Correction: 26 (65) [Prior] Fistula Closure: 30 (75) [Prior]”
Hirano 2001 [25]	30	58 (100)	-	28 (48)	Genioplasty: 18 (31) [Prior]
Trindade 2003 [27]	9	29 (100)	-		-
Janulewicz 2004 [29]	3–6	54 (100)	-	20 (37)	-
Niemeyer 2005 [30]	3–12	42 (100)	-	-	-
Thongdee 2005 [31]	62	30 (100)	-	11 (37)	Genioplasty: 4 (13) [Concurrent]
Wolford 2008 [32]	44	12 (100)	-	5 (42)	-
Kim 2012 [33]	12	8 (100)	-	-	-
Kumari 2013 [34]	29	9 (100)	-	-	Bone Graft: 9 (100) [Prior]
Pereira 2013 [35]	12	20 (100)	-	-	-
Davidson 2014 [36]	24	11 (100)	-	-	-
Karabekmez 2015 [38]	66	15 (100)	-	9 (60)	“Pharyngoplasty/Flap: 4 (27) [Prior] Bone Graft: 12 (80) [Prior]”
Park 2015 [39]	6	25 (100)	-	25 (100)	-
Wu 2015 [40]	10.34	47 (100)	-	47 (100)	-
Chang 2017 [41]	6	18 (100)	-	18 (100)	-
Impieri 2018 [42]	12	47 (100)	-	17 (36)	Pharyngoplasty/Flap: 18 (38) [Prior]
Jeong 2018 [43]	13.87	41 (100)	-	-	-
Alaluusua 2019 [44]	8.7	100 (100)	-	-	“Pharyngoplasty/Flap: 19 (19) [Prior] Palatoplasty: 6 (6) [Prior]”
Hagberg 2019 [46]	12	15 (100)	-	9 (60)	Palatoplasty: 15 (100)
Harjunpää 2019 [47]	8.7	93 (100)	-	24 (26)	“Pharyngoplasty/Flap: 25 (27) [Prior] Palatoplasty: 12 (13) [Prior]”
Schultz 2019 [48]	4.64	18 (100)	-	4 (22)	“Pharyngoplasty/Flap: 7 (39) [Prior] Bone Graft: 7 (39) [Concurrent] Genioplasty: 1 (6) [Concurrent] Palatoplasty: 3 (17) [Prior] Fistula Closure: 5 (28) [Concurrent] Bony Nasal Septoplasty: 4 (22) [Concurrent] Inferior Turbinectomy: 4 (22) [Concurrent]”
Alaluusua 2020 [50]	8.7	59 (100)	-	-	“Pharyngoplasty/Flap: 13 (22) [Prior] Palatoplasty: 5 (8) [Prior]”
Ganske 2020 [51]	6	19 (100)	-	-	-
Pereira 2020 [52]	12	20 (100)	-	7 (35)	Pharyngoplasty/Flap: 1 (5) [Prior]
Saleh 2020 [53]	0.7	23 (100)	-	23 (100)	-
Susarla 2020 [31(5)] [54]	12	28 (100)	-	8 (29)	Bone Graft: 14 (50) [Concurrent]
Susarla 2020 [49(4)] [55]	12	25 (100)	-	-	-
Ho 2021 [56]	12	17 (100)	-	-	-
Parikh 2021 [57]	12	35 (100)	-	-	-
Harjunpää 2022 [59]	8.7	57 (100)	-	18 (32)	“Pharyngoplasty/Flap: 11 (19) Genioplasty: 2 (4) Palatoplasty: 3 (5)”
Jang 2022 [69]	6	19 (100)	-	19 (100)	-
Seixas 2022 [60]	17	535 (100)	-	240 (45)	-
Tekin 2022 [61]	63	10 (100)	-	-	Bone Graft: 10 (100) [Prior]
Tsang 2022 [62]	12	17 (100)	-	-	-
May 2023 [67]	6	10 (100)	-	-	-
Idso 2023 [64]	2–3.2	30 (100)	-	-	-
Liao 2023 [65]	18	35 (100)	-	35 (100)	Genioplasty: 12 (34) [Concurrent]
Su 2023 [68]	6	162 (100)	-	162 (100)	Pharyngoplasty/Flap: 43 (27) [Prior]

**Table 5:** Methodology of included studies with comparator groups (12 studies).

	Follow-Up Period (Average Months)	Study Groups	“Patients per Group”	Le Fort I # (%)	Le Fort II # (%)	“Bimaxillary Osteotomy # (%)”	Prior/Concurrent Procedures # (%)
Cheung 1994 [17]	28	UCLP	30	30 (100)	-	14 (47)	Bone Graft: 30 (100) [Concurrent]
		BCLP	16	16 (100)	-	9 (56)	Bone Graft: 16 (100) [Concurrent]
Ayliffe 1995 [20]	12	Fixation	25	25 (100)	-	11 (44)	Bone Graft: 8 (32) [Concurrent]
		Plated	36	36 (100)	-	15 (42)	Bone Graft: 30 (83) [Concurrent]
		All patients	61	61 (100)	-	26 (43)	Bone Graft: 38 (62) [Concurrent]
Stewart 1996 [21]	12	BCLP	6	6 (100)	-	-	Bone Graft: 6 (100) [Prior]
		UCLP	13	13 (100)	-	-	Bone Graft: 13 (100)
		CP	5	5 (100)	-	-	-
Heliövaara 2002 [26]	12	CP	14	14 (100)	-	-	“Pharyngoplasty/Flap: 3 (21) [Prior] Rhinoplasty: 2 (14) [Prior]”
		BCLP	11	11 (100)	-	-	“Pharyngoplasty/Flap: 2 (18) [Prior] Bone Graft: 11 (100) [5 Prior; 6 Concurrent] Rhinoplasty: 11 (100) [Prior] Lip Correction: 11 (100) [Prior] Fistula Closure: 30 (75) [Prior]”
Heliövaara 2004 [28]	12	UCLP	30	30 (100)	-	-	“Bone Graft: 27 (90) [Prior] Rhinoplasty: 25 (83) [Prior] Lip Correction: 19 (63) [Prior] Fistula Closure: 23 (77) [Prior]”
		BCLP	9	9 (100)	-	-	“Bone Graft: 7 (78) [Prior] Rhinoplasty: 9 (100) [Prior] Lip Correction: 9 (100) [Prior]”
		CP	11	11 (100)	-	-	-
Watts 2014 [37]	21.6	1-piece LF1	11	11 (100)	-	6 (55)	“Bone Graft: 11 (100) [Concurrent] Fistula Closure: 2 (18) [Concurrent]”
		2-piece LF1	19	19 (100)	-	10 (53)	“Bone Graft: 19 (100) [Concurrent] Fistula Closure: 4 (21) [Concurrent]”
Watts 2015 [5]	21	Planned Movements >10 mm	10	10 (100)	-	-	Bone Graft: 10 (100)
		Planned Movements <10 mm	20	20 (100)	-	-	Bone Graft: 20 (100)
De Medeiros-Santana 2019 [45]	14	(-) Hypernasality	41	41 (100)	-	-	-
		(+) Hypernasality	11	11 (100)	-	-	-
Yatabe-Ioshida 2019 [49]	12	UCLP	9	NR	NR	9 (100)	-
		BCLP	6	NR	NR	6 (100)	-
Wangsrimongkol 2021 [58]	12	Minor Advancement: <5 mm	6	6 (100)	-	-	“Pharyngoplasty/Flap: 1 (17) [Prior] Bone Graft: 2 (33) [Concurrent] Genioplasty: 1 (17) [Concurrent]”
		Moderate Advancement: 5–10 mm	30	30 (100)	-	-	“Pharyngoplasty/Flap: 1 (3) [Prior] Bone Graft: 7 (23) [Concurrent] Genioplasty: 3 (10) [Concurrent]”
		Major Advancement: >10 mm	13	13 (100)	-	-	“Pharyngoplasty/Flap: 2 (15) [Prior] Bone Graft: 2(15) [Concurrent] Genioplasty: 2(15) [Concurrent]”
Wangsrimongkol 2022 [63]	15.8	Mild Cleft-Maxillary Hypoplasia	14	14 (100)	-	-	-
		Moderate Cleft-Maxillary Hypoplasia	29	29 (100)	-	-	-
		Severe Cleft-Maxillary Hypoplasia	8	8 (100)	-	-	-
Mansour 2023 [66]	28.9	Conventional Osteotomy	14	14 (100)	-	14 (100)	-
		Modified	10	10 (100)	-	10 (100)	-

**Table 6:** Summary of VP, speech, and airway outcomes (34 studies).

Number of Studies	Reported Outcome	Results Overall (Average Change)
8	VP Insufficiency/Function	63% worsening
8	Pharyngeal/Airway Dimensions (Area and Volume)	13% increased
5	Pharyngeal Depths	17% increased
10	Resonance/Hypernasality	15% worsening
5	Speech Articulation	33% improve
4	Nasal Emission/Turbulence	20% worsening

VP: velopharyngeal.

**Table 7:** Summary of cephalometric outcomes (31 studies).

Vertical and Horizontal Movements			
Number of Studies	Reported Outcome	Mean Surgical Change	
26	Horizontal advance (mm)	6.09	
11	Le Fort I	6.58	
4	Bimaxillary Osteotomy	3.95	
23	Horizontal relapse (mm)	−0.98	
10	Le Fort I	−0.97	
2	Bimaxillary Osteotomy	−0.52	
23	Vertical advance (mm)	3.28	
10	Le Fort I	3.07	
3	Bimaxillary Osteotomy	3.26	
21	Vertical relapse (mm)	−0.89	
9	Le Fort I	−0.74	
2	Bimaxillary Osteotomy	−0.86	
Angular Movement			
Number of Studies	Reported Outcome	Mean Surgical Change	Mean Relapse
14	SNA Angle (°)	5.88	−1.27
8	Le Fort I	6.11	−1.13
1	Bimaxillary Osteotomy	3.77	-
1	Le Fort II	6.10	−1.60
5	SNB Angle (°)	−1.27	0.90
4	Le Fort I	−1.32	0.96
1	Le Fort II	−0.80	0.40
6	ANB Angle (°)	7.54	−3.51
4	Le Fort I	7.82	−3.22
1	Le Fort II	5.00	−6.00

°: degrees; mm: milimeter.

## Supplementary Materials

The following supporting information can be downloaded at: https://www.mdpi.com/article/10.3390/jcm13195703/s1, Table S1: Bias and Quality Assessment; Table S2: Reported Velopharyngeal and Soft Tissue Outcomes; Table S3: Reported Speech and Airway Outcomes; Table S4: Reported Cephalometric Outcomes: Angular Movement; Table S5: Reported Cephalometric Outcomes: Vertical Movement; Table S6: Reported Cephalometric Outcomes: Horizontal Movement.
