# Supplementary material for: Reshaping Faces, Redefining Risks: A Systematic Review of Orthognathic Surgery Outcomes in Cleft Lip and Palate Patients
Source: J Clin Med. 2024 Sep 25;13(19):5703. doi: 10.3390/jcm13195703 (PMC11477402; doi:10.3390/jcm13195703)
Supplement: Supplementary file 1 [file jcm-13-05703-s001.zip › jcm-3167417-supplementary.pdf]

Supplementary Table S1. Bias and Quality Assessment (Cochrane ROBINS-I)

	D1	D2	D3	D4	D5	D6	D7	OVERALL
Schendel 1979 [9]	Green	Green	Green	Green	Green	Green	Green	Green
Ward-Booth 1984 [10]	Yellow	Green	Green	Green	Green	Green	Green	Yellow
Adlam 1989 [11]	Yellow	Green	Green	Green	Green	Green	Green	Yellow
Houston 1989 [12]	Yellow	Green	Green	Green	Green	Green	Green	Yellow
Posnick 1990 [13]	Green	Green	Green	Green	Green	Green	Green	Green
Watzke 1990 [14]	Yellow	Green	Green	Green	Green	Yellow	Green	Yellow
Hochban 1993 [15]	Green	Green	Green	Green	Green	Green	Green	Green
Okazaki 1993 [16]	Yellow	Green	Green	Green	Green	Yellow	Green	Yellow
Cheung 1994 [17]	Green	Green	Green	Green	Green	Green	Green	Green
Posnick 1994 [94(1)] [18]	Green	Green	Green	Green	Green	Green	Green	Green
Posnick 1994 [94(7)] [19]	Green	Green	Green	Green	Green	Green	Green	Green
Ayliffe 1995 [20]	Yellow	Green	Green	Green	Green	Green	Green	Yellow
Stewart 1996 [21]	Green	Green	Green	Green	Green	Green	Green	Green
Haapanen 1997 [22]	Yellow	Green	Green	Green	Green	Yellow	Green	Yellow
Maegawa 1998 [23]	Yellow	Green	Green	Green	Green	Yellow	Green	Yellow
Heliövaara 2001 [24]	Yellow	Green	Green	Green	Green	Green	Green	Yellow
Hirano 2001 [25]	Yellow	Green	Green	Green	Green	Green	Green	Yellow
Heliövaara 2002 [26]	Green	Green	Green	Green	Green	Green	Green	Green
Trindade 2003 [27]	Yellow	Green	Green	Green	Green	Yellow	Green	Yellow
Heliövaara 2004 [28]	Green	Green	Green	Green	Green	Green	Green	Green
Janulewicz 2004 [29]	Yellow	Green	Green	Green	Green	Yellow	Green	Yellow
Niemeyer 2005 [30]	Yellow	Green	Green	Green	Green	Yellow	Green	Yellow
Thongdee 2005 [31]	Green	Green	Green	Green	Green	Green	Green	Green
Wolford 2008 [32]	Yellow	Green	Green	Green	Green	Green	Green	Yellow
Kim 2012 [33]	Green	Green	Green	Green	Green	Yellow	Green	Yellow
Kumari 2013 [34]	Yellow	Green	Green	Green	Green	Green	Green	Yellow
Pereira 2013 [35]	Green	Green	Green	Green	Green	Yellow	Green	Yellow
Davidson 2014 [36]	Yellow	Green	Green	Green	Green	Green	Green	Yellow
Watts 2014 [37]	Green	Green	Green	Green	Green	Green	Green	Green
Karabekmez 2015 [38]	Yellow	Green	Green	Green	Green	Green	Green	Yellow
Park 2015 [39]	Yellow	Green	Green	Green	Green	Green	Green	Yellow
Watts 2015 [40]	Green	Green	Green	Green	Green	Green	Green	Green
Wu 2015 [41]	Yellow	Green	Green	Green	Green	Green	Green	Yellow
Chang 2017 [42]	Green	Green	Green	Green	Green	Green	Green	Green
Impieri 2018 [43]	Yellow	Green	Green	Green	Green	Yellow	Green	Yellow
Jeong 2018 [44]	Yellow	Green	Green	Green	Yellow	Green	Green	Yellow

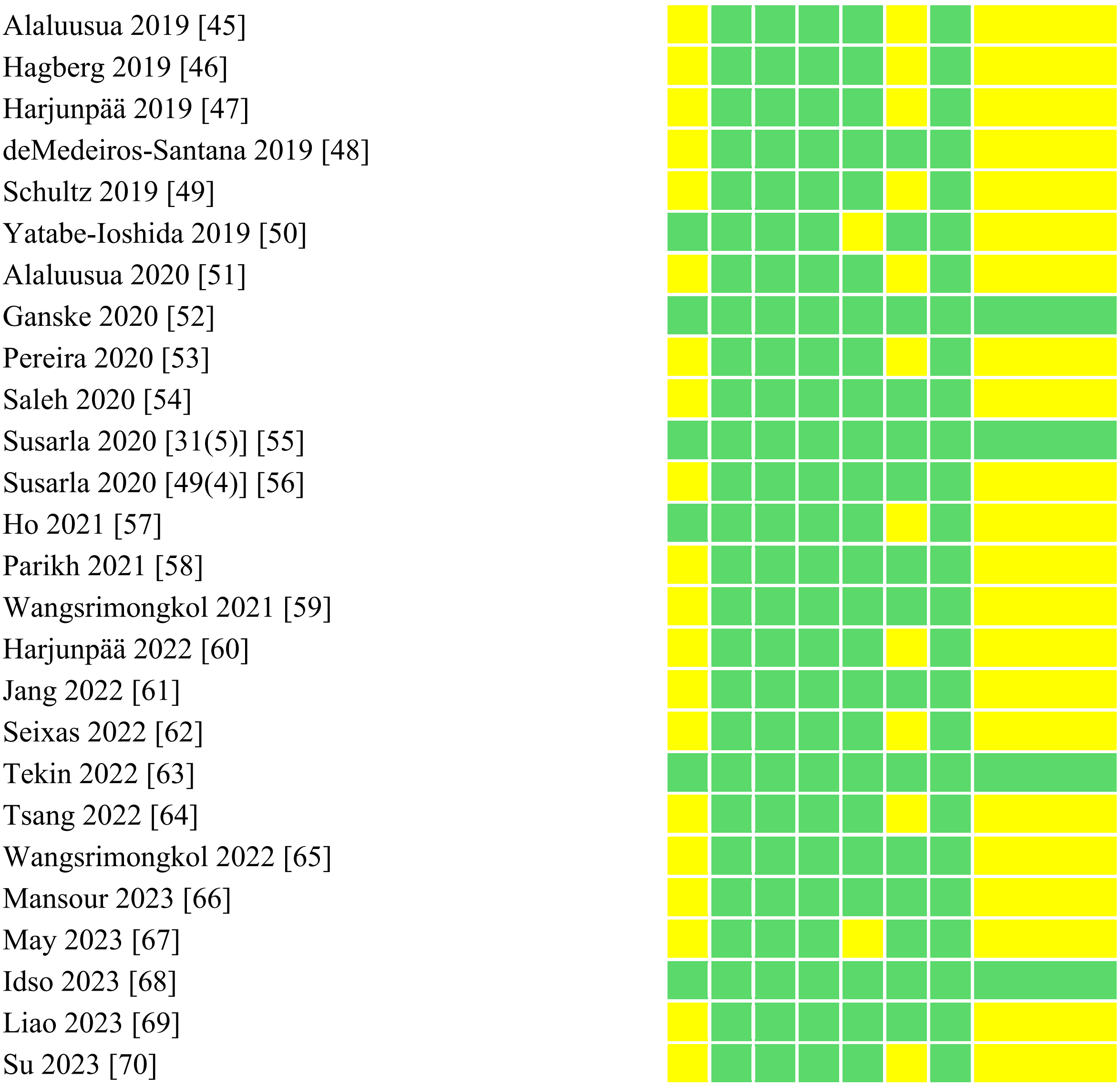

Low risk of bias
Moderate risk of bias
High risk of bias

Domains:

D1: Bias due to confounding.

D2: Bias due to selection of participants.

D3: Bias in classification of interventions.

D4: Bias due to deviations from intended interventions.

D5: Bias due to missing data.

D6: Bias in measurement of outcomes.

D7: Bias in selection of the reported result.

Table S2. Reported velopharyngeal and soft tissue outcomes (23 studies)

	Post-op Period (average months)	Reported Outcome	Result
Schendel 1979 [9]	26	Soft Palate Angulation	Increased angulation (129º to 138.1º)
Schendel 1979 [9]	26	Soft Palate Length	Increased length (29.8 mm to 31.6 mm)
Schendel 1979 [9]	26	Functional Pharyngeal Length	Decreased length (14.3 mm to 13.8 mm)
Schendel 1979 [9]	26	Pharyngeal Depth	Increased depth (22.6 mm to 23 mm)
Schendel 1979 [9]	26	Posterior Pharyngeal Wall Thickness	Decreased thickness (6.9 mm to 6.8 mm)
Watzke 1990 [14]	12	VP Function	Increased severity score (1.75 to 2.08) [1=adequate; 4=inadequate]
Watzke 1990 [14]	12	VP Orifice Area	Increased mean change (+0.094 cm^2)
Okazaki 1993 [16]	12	Pharyngeal Depth	Increased depth (24.63 mm to 27 mm)
Okazaki 1993 [16]	12	Velar Length	Increased length (26.25 mm to 26.38 mm)
Haapanen 1997 [22]	12	VP Insufficiency	Increased insufficiency score (1.53 to 1.8) [1=competent; 3=insufficient]
Haapanen 1997 [22]	12	VP Orifice Area	Increased area (3 mm^2 to 3.1 mm^2)
Haapanen 1997 [22]	12	Nasal Cross-Sectional Area	Increased area (38.9 mm^2 to 48.8 mm^2)**
Trindade 2003 [27]	9	VP Orifice Area	Decreased area (21 mm^2 to 17 mm^2)
Trindade 2003 [27]	9	Nasal Cross-Sectional Area	Increased area (34 mm^2 to 46 mm^2)
Heliövaara 2004 [28]	12	Lower Sagittal Depth of the Nasopharyngeal Airway	Increased depth among all groups (UCLP**: 20.1 mm to 24.1 mm; BCLP*: 22.4 mm to 26.2 mm; CP**: 22.6 mm to 26.3 mm)
Heliövaara 2004 [28]	12	Upper Sagittal Depth of the Nasopharyngeal Airway	Increased depth among all groups (UCLP**: 19.2 mm to 23.1 mm; BCLP**: 19.8 mm to 23.4 mm; CP*: 21.8 mm to 24.9 mm)

Janulewicz 2004 [29]	3-6	VP Function	Increased insufficiency score (1.56 to 2.44) [1=competent; 4=complete VPI]
Pereira 2013 [35]	12	Velopharyngeal Composite Score	Increased composite score (0.22 to 0.72) [increased nasality, turbulence, emission]
Davidson 2014 [36]	5.1	Nasal Interalar Width	Increased mean width (+1.9 mm)*
Davidson 2014 [36]	5.1	Internostril Width	Decreased mean width (-0.2 mm)*
Davidson 2014 [36]	5.1	Nasal Tip Projection	Decreased mean projection (-1 mm)
Davidson 2014 [36]	5.1	Columellar Length	Decreased mean length (-0.7 mm)**
Davidson 2014 [36]	5.1	Nasal Labial Angle	Decreased mean angulation (-0.2º)
Davidson 2014 [36]	5.1	Nasal Length	Decreased mean length (-0.7 mm)
Park 2015 [39]	6	Change in Soft Tissue - Interlabial Gap	Increased (+0.91 mm)
Park 2015 [39]	6	Change in Soft Tissue - Labrale Superius	Increased (+0.7 mm)
Park 2015 [39]	6	Change in Soft Tissue - Superus Media	Increased (+0.12 mm)
Park 2015 [39]	6	Change in Soft Tissue - Inferus Media	Decreased (-0.04 mm)
Park 2015 [39]	6	Change in Soft Tissue - Labrale Inferius	Increased (+0.29 mm)
Park 2015 [39]	6	Change in Soft Tissue - Inferior Labial Sulcus	Decreased (-0.23 mm)
Park 2015 [39]	6	Change in Soft Tissue - Pogonion	Decreased (-0.01 mm)
Park 2015 [39]	6	Change in Soft Tissue - Menton	Increased (+0.08 mm)
Wu 2015 [41]	10.34	Nasopharyngeal Depth	Increased depth (27.89 mm to 32.55 mm)**
Wu 2015 [41]	10.34	Oropharyngeal Depth	Decreased depth (13.5 mm to 13.3 mm)
Wu 2015 [41]	10.34	Velar Thickness	Decreased thickness (10.57 mm to 10.1 mm)**
Wu 2015 [41]	10.34	Velar Angle	Increased angulation (133.4º to 141.5º)**

Wu 2015 [41]	10.34	Thickness of Posterior Pharyngeal Wall	Increased thickness (4.62 mm to 5.21 mm)
Alaluusua 2019 [45]	8.7	VP Insufficiency	Increased severity score (1.43 to 1.88) [1=competent; 5=severe VPI]
Harjunpää 2019 [47]	8.05	VP Insufficiency	Increased severity score (0.44 to 0.91) [0=normal; 4=severe]
Harjunpää 2019 [47]	8.05	Oropharynx - Horizontal Change	Increased (15.8 mm to 16.6 mm)
Harjunpää 2019 [47]	8.05	Upper Hypopharnx - Horizontal Change	Decreased (11.3 mm to 10.9 mm)
deMedeiros-Santana 2019 [48]	14	Palatal Length	(+) Hypernasality average score: 1.8; (-) Hypernasality average score: 2.5 [1=long; 2=regular; 3= short]
deMedeiros-Santana 2019 [48]	14	Levator Veli Palatini Mobility	(+) Hypernasality average score: 1.6; (-) Hypernasality average score: 2.2 [1=good; 2=regular; 3= poor]
deMedeiros-Santana 2019 [48]	14	Levator Veli Palatini Insertion	(+) Hypernasality average score: 1.3; (-) Hypernasality average score: 2.2 [1=posterior; 2=middle; 3= anterior]
Schultz 2019 [49]	4.64	VP Function	Increased severity score (1.16 to 1.61) [0=competent; 2=incompetent]
Schultz 2019 [49]	4.64	VP Closure	Decreased closure score (0.07 to 0.3) [1=inadequate closure >0.05 cm^2]
Yatabe-Ioshida 2019 [50]	12	Airway Cross-Sectional Area	Increased area among both groups (UCLP: 191.36 mm^2 to 204.73 mm^2; BCLP: 224.83 mm^2 to 220.37 mm^2)
Yatabe-Ioshida 2019 [50]	12	Nasal Cavity Airway Dimension	Increased volume among both groups (UCLP: 13.71 cm^3 to 13.96 cm^3; BCLP: 11.24 cm^3 to 12.39 cm^3)
Yatabe-Ioshida 2019 [50]	12	Nasopharynx Airway Dimension	Increased volume among both groups (UCLP: 15.77 cm^3 to 18.9 cm^3; BCLP: 22.18 cm^3 to 26.27 cm^3)
Yatabe-Ioshida 2019 [50]	12	Oropharynx Airway Dimension	UCLP: Increased 7.46 cm^3 to 7.91 cm^3; BCLP: Decreased 9.4 cm^3 to 9.09 cm^3
Ganske 2020 [52]	6	Columellar Length	Decreased mean length (-1.07 mm)**
Ganske 2020 [52]	6	Columellar Width	Increased mean width (+0.2 mm)
Ganske 2020 [52]	6	Overall Nasal Width	Increased mean width (+1.94 mm)**

Saleh 2020 [54]	0.01	Airway Cross-Sectional Area	(+) Prior CP Repair: Increased (416.9 mm^2 to 424.8 mm^2); (-) Prior CP Repair: Decreased (329.5 mm^2 to 310.8 mm^2)
Saleh 2020 [54]	0.01	Airway Volume	(+) Prior CP Repair: Increased (3.9 cm^3 to 4.4 cm^3); (-) Prior CP Repair: Increased (4.2 cm^3 to 4.3 cm^3)
Seixas 2022 [62]	17	Nasal Cross-Sectional Area	Increased area (0.53 cm^2 to 0.64 cm^2)
Seixas 2022 [62]	17	Airway Cross-Sectional Area	Increased area (0.89 cm^2 to 0.91 cm^2)
Tekin 2022 [63]	63	Superior Posterior Pharyngeal Depth	Increased depth (8.8 mm to 11.9 mm)*
Tekin 2022 [63]	63	Palatal Pharyngeal Depth	Increased depth (21.4 mm to 28.5 mm)*
Tekin 2022 [63]	63	Middle Pharyngeal Depth	Increased depth (7.6 mm to 10.2 mm)*
Tekin 2022 [63]	63	Inferior Pharyngeal Depth	Increased depth (8.7 mm to 9.1 mm)
Tekin 2022 [63]	63	Epiglottic Pharyngeal Depth	Increased depth (8.4 mm to 9.6 mm)
Tekin 2022 [63]	63	Sagittal Cross-Sectional Area of the Nasopharynx (CSAN)	Increased area (136.7 mm^2 to 231.9 mm^2)*
Tekin 2022 [63]	63	Sagittal Cross-Sectional Area of the Velopharynx (CSAV)	Increased area (185 mm^2 to 252.4 mm^2)**
Tekin 2022 [63]	63	Sagittal Cross-Sectional Area of the Oropharynx (CSAO)	Increased area (293.9 mm^2 319.3 mm^2)
Tekin 2022 [63]	63	Total Area: sum of CSAN, CSAV and CSAO	Increased area (615.6 mm^2 to 803.6 mm^2)**
May 2023 [67]	6	Pre-Genu Palatal Measurement (resting)	Increased (9.1 mm to 11.9 mm)*
May 2023 [67]	6	Pre-Genu Palatal Measurement (closed)	Increased (9.9 mm to 12.7 mm)*
May 2023 [67]	6	Post-Genu Palatal Measurement (resting)	Increased (9.2 mm to 10 mm)
May 2023 [67]	6	Post-Genu Palatal Measurement (closed)	Increased (8.8 mm to 9.1 mm)

May 2023 [67]	6	Genu Angle (resting)	Decreased angulation (129.3º to 116.3º)
May 2023 [67]	6	Genu Angle (closed)	Decreased angulation (115.5º to 99.2º)*
Idso 2023 [68]	2-3.2	Airway Volume	Increased volume (19121 mm^3 to 21750 mm^3)
Su 2023 [70]	6	VP Insufficiency	Increased insufficiency (19.8% to 28.4% patients)

* = P value <.05; ** = P value <.01. No asterisk indicates non significant P-value or no P value given

VP: velopharyngeal; VPI: velopharyngeal insufficiency; CP/SCMP: cleft palate/submucous cleft palate; UCLP: unilateral cleft palate; BCLP: bilateral cleft palate; CP: cleft palate; NR: Not reported

Table S3. Reported speech and airway outcomes (18 studies)

	Post-op Period (average months)	Reported Outcome	Result
Okazaki 1993 [16]	12	Nasal Emission	Increased severity score (1.9 to 2.1) [1=absent; 3=severe]
Okazaki 1993 [16]	12	Hypernasality	Increased severity score (2.7 to 3.6) [1=absent; 5=severe]
Haapanen 1997 [22]	12	Nasalance	Increased percentage of patients (22 to 26)*
Haapanen 1997 [22]	12	Hypernasality	Increased percentage of patients (27 to 47)
Maegawa 1998 [23]	8.4	Speech Intelligibility	Increased unintelligibility score (1.75 to 1.9) [1=intelligible; 4=severe unintelligible]
Trindade 2003 [27]	9	Nasalance	Increased percentage of patients (43 to 52)*

Janulewicz 2004 [29]	3-6	Speech Articulation	Increased mean speech score (2.46 to 4.24)
Janulewicz 2004 [29]	3-6	Hypernasality	Increased severity score (1.61 to 1.74) [1=normal; 4=severe]
Janulewicz 2004 [29]	3-6	Hyponasality	Decreased percentage of patients (33 to 15)
Niemeyer 2005 [30]	3-12	Resonance	Percentage of patients (increase to 47.5)
Niemeyer 2005 [30]	3-12	Hypernasality	Increase percentage of patients (75 to 87.5)
Kim 2012 [33]	8	Speech Articulation	No change
Kim 2012 [33]	8	Hypernasality	No change
Pereira 2013 [35]	12	Resonance	Decreased number of patients (16 to 11)
Pereira 2013 [35]	12	Nasal Turbulence	Incrased number of patients (3 to 11)
Pereira 2013 [35]	12	Hypernasality	Increased number of patients (4 to 9)
Chang 2017 [42]	6	Apnea Hypopnea Index (AHI)	Decreased AHI score (1.99 to 1.86)
Impieri 2018 [43]	12	Nasal Emission	No change
Impieri 2018 [43]	12	Nasal Turbulence	Increased severity score (0.13 to 0.17 [0=absent; 1=mild and consistent]
Impieri 2018 [43]	12	Hypernasality	Increased severity score (0.15 to 0.25) [0=absent; 1=mild and consistent]
Impieri 2018 [43]	12	Hyponasality	Decreased severity score (0.27 to 0.14) [0=absent; 1=mild and consistent]
Hagberg 2019 [46]	12	Oral Consonents Correct	Increased percentage (82 to 95)**
Hagberg 2019 [46]	12	Correct Articulation of /s/	Increased percentage (34 to 85)**
Schultz 2019 [49]	4.64	Nasal Emission	Increased percentage of patients (72.2 to 88.8)
Schultz 2019 [49]	4.64	Hypernasality	Increased severity score (1.94 to 2.16) [0=normal; 4=severe]
Alaluusua 2020 [51]	8.7	Speech Articulation	Decreased percentage of patients with misarticulation of 1-3 of the alveolar sounds /s/, /l/, or /r/ (63 to 20)

Pereira 2020 [53]	12	Nasalance	Increased percentage of patients (23 to 31)**
Ho 2021 [57]	12	Vowel Height	Increased mean change (+1.09)
Ho 2021 [57]	12	Vowel Frontness	Increased mean change (+1.27)
Harjunpää 2022 [60]	8.7	Articulation Errors of /l/	Decreased number of patients (19 to 11)
Harjunpää 2022 [60]	8.7	Articulation Errors of /r/	Decreased number of patients (26 to 23)
Harjunpää 2022 [60]	8.7	Speech Articulation	Decreased number of patients with articulation errors of /s/ (18 to 13)
Seixas 2022 [62]	17	Nasal Obstruction	Decreased percentage of patients (44.8 to 26.7)
Seixas 2022 [62]	17	Oronasal Breathing	Decreased percentage of patients (72.1 to 49.8)
Seixas 2022 [62]	17	Snoring	Decreased percentage of patients (33.7 to 21.3)
Seixas 2022 [62]	17	Sleep Respiratory Obstruction	Decreased percentage of patients (6.2 to 3)
Tsang 2022 [64]	12	Spectral Moments of /s/: Center of Gravity	Increased Hz (5230 to 5850)
Tsang 2022 [64]	12	Spectral Moments of /s/: Standard Deviation	Decreased Hz (1977 to 1860)
Tsang 2022 [64]	12	Spectral Moments of /s/: Skewness	Decreased (0.15 tp -0.24)
Tsang 2022 [64]	12	Spectral Moments of /s/: Kurtosis	Decreased (1.14 to 0.89)
Tsang 2022 [64]	12	Spectral Moments of /f/: Center of Gravity	Increased Hz (3823 to 4397)
Tsang 2022 [64]	12	Spectral Moments of /f/: Standard Deviation	Increased Hz (2218 to 2390)
Tsang 2022 [64]	12	Spectral Moments of /f/: Skewness	Decreased (1.35 to 0.8)
Tsang 2022 [64]	12	Spectral Moments of /f/: Kurtosis	Decreased (3.75 to 1.56)

* = P value <.05; ** = P value <.01. No asterisk indicates non significant P-value or no P value given

Hz: hertz

Table S4. Reported cephalometric outcomes: angular movement (14 studies)

	Post-op Period (average months)	Group 1 Surgical Change	Group 1 Relapse	Group 2 Surgical Change	Group 2 Relapse	Group 3 Surgical Change	Group 3 Relapse
SNA Angle (Degrees)							
Ward-Booth 1984 [10]	22	6.10*	-1.60*	-	-	-	-
Hochban 1993 [15]	12	5.20**	-1.40*	-	-	-	-
Heliövaara 2001 [24]	12	4.20	-0.70	-	-	-	-
Heliövaara 2002 [26]	12	4.80**	-0.30	4.8*	-0.80	-	-
Thongdee 2005 [31]	62	3.95	-2.15	-	-	-	-
Wolford 2008 [32]	44	-	-3.33*	-	-	-	-
Watts 2014 [37]	12	6.00**	-1.00	6.20**	-2.10	-	-
Watts 2015 [40]	12	8.40**	-1.80**	4.00**	-1.70**	-	-
Jeong 2018 [44]	14	2.29	-	-	-	-	-
Susarla 2020 [31(5)] [55]	12	6.00	0.80	-	-	-	-
Susarla 2020 [49(4)] [56]	12	6.80	-0.70	-	-	-	-
Wangsrimongkol 2021 [59]	12	3.90	-1.30	7.30	-1.00	10.10	-1.50
Jang 2022 [61]	6	3.77					
Wangsrimongkol 2022 [65]	15.8	6.20**	-0.90*	7.70**	-1.20**	9.80**	-1.40**
SNB Angle (Degrees)							
Ward-Booth 1984 [10]	22	-0.80	0.40	-	-	-	-
Heliövaara 2001 [24]	12	-2.20*	0.70	-	-	-	-
Heliövaara 2002 [26]	12	-1.40	0.90	-3.20	0.80	-	-
Wangsrimongkol 2021 [59]	12	-2.00*	1.10	-0.90	1.30	0.00	0.70

Wangsrimongkol 2022 [65]	15.8	-0.90	1.20**	-0.90**	1.20**	-0.40**	0.70*
ANB Angle (Degrees)							
Ward-Booth 1984 [10]	22	5.00*	-6.00*	-	-	-	-
Heliövaara 2001 [24]	12	6.40	-5.70*	-	-	-	-
Heliövaara 2002 [26]	12	6.20	-4.70	8.00	-5.00	-	-
Wolford 2008 [32]	44	-	-3.58**	-	-	-	-
Wangsrimongkol 2021 [59]	12	5.80	-2.40	8.20	-2.30	10.10	-2.20
Wangsrimongkol 2022 [65]	15.8	7.00**	-2.10*	8.60**	-2.50**	10.10**	-2.10**

* = P value <.05; ** = P value <.01. No asterisk indicates non significant P-value or no P value given

Table S5. Reported cephalometric outcomes: vertical movement (23 studies)

	Post-op Period (average months)	Group 1 Mean Change	Group 2 Mean Change	Group 3 Mean Change
Vertical advance (mm):				
Adlam 1989 [11]	22	5.70	-	-
Houston 1989 [12]	17	3.30	-	-
Posnick 1990 13]	24	2.60	-	-
Cheung 1994 [17]	28	4.23	3.09	-
Posnick 1994 [94(1)] [18]	12	2.00	-	-
Posnick 1994 [94(7)] [19]	12	2.10	-	-
Ayliffe 1995 [20]	12	5.00	6.00	5.70
Stewart 1996 [21]	3	2.20	2.60	-1.00
Heliövaara 2002 [26]	12	3.60	7.30	-
Heliövaara 2004 [28]	12	4.70	6.30	4.20
Thongdee 2005 [31]	62	4.40	-	-
Kumari 2013 [34]	12	5.21	-	-
Watts 2014 [37]	12	2.70**	2.70**	-
Karabekmez 2015 [38]	66	3.90	-	-

Watts 2015 [40]	12	2.30**	3.10**	-
Susarla 2020 [31(5)] [55]	12	0.02	-	-
Susarla 2020 [49(4)] [56]	12	2.10	-	-
Parikh 2021 [58]	12	0.96	-	-
Wangsrimongkol 2021 [59]	12	2.80	2.10	2.50
Jang 2022 [61]	6	1.80	-	-
Wangsrimongkol 2022 [65]	12	2.10**	2.10**	2.90
Mansour 2023 [66]	29	4.55	5.67	-
Liao 2023 [69]	18	1.00**	-	-
Vertical relapse (mm):				
Adlam 1989 [11]	22	-1.55	-	-
Houston 1989 [12]	17	-0.08	-	-
Posnick 1990 13]	24	-1.40	-	-
Cheung 1994 [17]	28	-0.95	-0.21	-
Posnick 1994 [94(1)] [18]	12	-0.60	-	-
Posnick 1994 [94(7)] [19]	12	-0.40	-	-
Ayliffe 1995 [20]	12	-1.50	-1.00	-1.20
Heliövaara 2002 [26]	12	-0.60	-1.30	-
Heliövaara 2004 [28]	12	-1.00	-1.12	-0.70
Thongdee 2005 [31]	62	-2.25	-	-
Kumari 2013 [34]	12	-2.01	-	-
Watts 2014 [37]	12	-0.60**	-1.50**	-
Karabekmez 2015 [38]	66	-1.10	-	-
Watts 2015 [40]	12	-0.50	-1.70**	-
Susarla 2020 [31(5)] [55]	12	-0.90	-	-
Susarla 2020 [49(4)] [56]	12	-0.80	-	-
Parikh 2021 [58]	12	-0.21	-	-
Wangsrimongkol 2021 [59]	12	-0.90	-0.30	-0.40
Wangsrimongkol 2022 [65]	12	-0.30	-0.40*	-0.20
Mansour 2023 [66]	29	-0.47	-1.41	-

Liao 2023 [69]	18	-0.70**	-	-

* = P value <.05; ** = P value <.01. No asterisk indicates non significant P-value or no P value given

mm: milimeter

Table S6. Reported cephalometric outcomes: horizontal movement (26 studies)

	Post-op Period (average months)	Group 1 Mean Change	Group 2 Mean Change	Group 3 Mean Change
Horizontal advance (mm):				
Adlam 1989 [11]	22	5.09	-	-
Houston 1989 [12]	17	9.00	-	-
Posnick 1990 13]	24	6.70	-	-
Cheung 1994 [17]	28	4.46	4.21	-
Posnick 1994 [94(1)] [18]	12	6.40	-	-
Posnick 1994 [94(7)] [19]	12	6.90	-	-
Ayliffe 1995 [20]	12	5.00	7.00	6.10
Stewart 1996 [21]	12	6.50	4.20	7.00
Hirano 2001 [25]	30	6.90	-	-
Heliövaara 2002 [26]	12	4.70	5.30	-
Heliövaara 2004 [28]	12	4.70	4.10	4.20
Thongdee 2005 [31]	62	5.64	-	-
Kumari 2013 [34]	12	5.17	-	-
Watts 2014 [37]	12	7.30**	7.50**	-
Karabekmez 2015 [38]	66	7.30	-	-
Watts 2015 [40]	12	9.80**	4.90**	-
Saleh 2020 [54]	0.7	6.18	-	-
Susarla 2020 [31(5)] [55]	12	6.10	-	-
Susarla 2020 [49(4)] [56]	12	5.80	-	-
Parikh 2021 [58]	12	7.05**	-	-
Wangsrimongkol 2021 [59]	12	4.10*	7.50*	11.30*
Jang 2022 [61]	6	5.61	-	-
Tekin 2022 [63]	63	9.22	-	-

Wangsrimongkol 2022 [65]	12	6.40**	8.10**	10.70**
Mansour 2023 [66]	29	2.01	0.63	-
Liao 2023 [69]	18	5.30**	-	-
Horizontal relapse (mm):				
Adlam 1989 [11]	22	-0.90	-	-
Houston 1989 [12]	17	-0.60	-	-
Posnick 1990 13]	24	-1.90	-	-
Cheung 1994 [17]	28	-0.99	-0.74	-
Posnick 1994 [94(1)] [18]	12	-1.00	-	-
Posnick 1994 [94(7)] [19]	12	-1.60	-	-
Ayliffe 1995 [20]	12	-0.50	-0.10	-0.30
Hirano 2001 [25]	30	-1.50	-	-
Heliövaara 2002 [26]	12	-0.40	-0.50	-
Heliövaara 2004 [28]	12	-0.57	-0.38	-0.36
Thongdee 2005 [31]	62	-1.77	-	-
Kumari 2013 [34]	12	-1.26	-	-
Watts 2014 [37]	12	-1.30	-1.90**	-
Karabekmez 2015 [38]	66	-1.90	-	-
Watts 2015 [40]	12	-1.80**	-1.50**	-
Susarla 2020 [31(5)] [55]	12	-0.80	-	-
Susarla 2020 [49(4)] [56]	12	-0.90	-	-
Parikh 2021 [58]	12	-0.79**	-	-
Wangsrimongkol 2021 [59]	12	-1.30	-1.00	-1.70
Tekin 2022 [63]	63	-2.00	-	-
Wangsrimongkol 2022 [65]	12	-1.00*	-1.30**	-1.50**
Mansour 2023 [66]	29	-0.75	-0.20	-
Liao 2023 [69]	18	-0.60**	-	-

* = P value <.05; ** = P value <.01. No asterisk indicates non significant P-value or no P value given

mm: milimeter

Search string:
(Cleft Palate [Mesh] OR Cleft Lip [Mesh] OR Orofacial Cleft [Mesh]) AND (Orthognathic Surgery [Mesh] OR Orthognathic Surgical Procedures [Mesh] OR Mandibular Osteotomy [Mesh] OR Maxillary Osteotomy [Mesh] OR Osteotomy, Sagittal Split Ramus [Mesh] OR Osteotomy, Le Fort [Mesh] OR Osteotomy [Mesh])

PICO Statement:

- **Population (P):** Patients with cleft lip and/or palate (CLP) undergoing orthognathic surgery following primary CLP repair
- **Intervention (I):** Orthognathic surgery following primary correction of the palate or lip
- **Comparator (C):** Patients with differing types of orthognathic surgery
- **Outcome (O):** Functional and structural outcomes, including velopharyngeal function, relapse rates, cephalometric changes, speech outcomes, and postoperative complications
